# Rabies outbreak in Brazil: first case series in children from an indigenous village

**DOI:** 10.1186/s40249-023-01130-y

**Published:** 2023-08-24

**Authors:** Dilceu Silveira Tolentino Júnior, Maryana Santos Vasconcelos Marques, Amanda Krummenauer, Magda Machado Saraiva Duarte, Silene Manrique Rocha, Mariana Gontijo de Brito, Ludmila Ferraz de Santana, Roberto Carlos de Oliveira, Eliseu Miranda de Assis, Kellyn Kessiene de Sousa Cavalcante, Carlos Henrique Alencar

**Affiliations:** 1 Postgraduate Program in Technology, Environment and Society, Federal University of the Jequitinhonha e Mucuri Valleys, Teófilo Otoni, MG Brazil; 2 Special Indigenous Health District of Minas Gerais and Espírito Santo, Ministry of Health, Governador Valadares, MG Brazil; 3 Brazil Field Epidemiology Training Program, Ministry of Health, Health and Environment Surveillance Secretariat, Brasília, DF Brazil; 4 Health Surveillance Secretariat, Ministry of Health, Brasília, DF Brazil; 5 State Department of Health of Minas Gerais, Coordination of Zoonoses, Belo Horizonte, MG Brazil; 6 Postgraduate Program in Integrated Territory Management, Vale do Rio Doce University, Governador Valadares, MG Brazil; 7 Federal Institute of Bahia, Eunápolis, BA Brazil; 8 Postgraduate Program in Public Health, Federal University of Ceará, Fortaleza, CE Brazil

**Keywords:** Rabies, Outbreak, Indigenous population, Low income, Brazil

## Abstract

**Background:**

Human rabies outbreak transmitted by bats continues to be a relevant public health problem not only in the Amazon region. The disease has affected one of the areas with the greatest poverty in southeastern Brazil, a region inhabited by the Maxakali indigenous people.

**Case presentation:**

We describe four cases of rabies among indigenous children that occurred in the indigenous village of Pradinho, municipality of Bertópolis, Minas Gerais, Brazil. Cases were notified between April and May 2022, all of whom died on average eight days after the first symptoms. All cases were observed in rural residents under 12 years of age. The probable form of exposure was through bat bites. The predominant symptoms were prostration, fever, dyspnea, sialorrhea, tachycardia, and altered level of consciousness. Half of the cases underwent late and/or incomplete post-exposure rabies prophylaxis, however, the other half underwent pre-exposure rabies prophylaxis, with only one case completing the scheme and another undergoing the adapted Milwaukee Protocol (Recife Protocol). All cases ended in death.

**Conclusions:**

This was the first rabies outbreak among indigenous people in Brazil. Among the manifested clinical forms in the series, there was a disease atypical presentation in at least one case. We suggest active surveillance and an intercultural educational campaign to prevent new cases.

**Graphical abstract:**

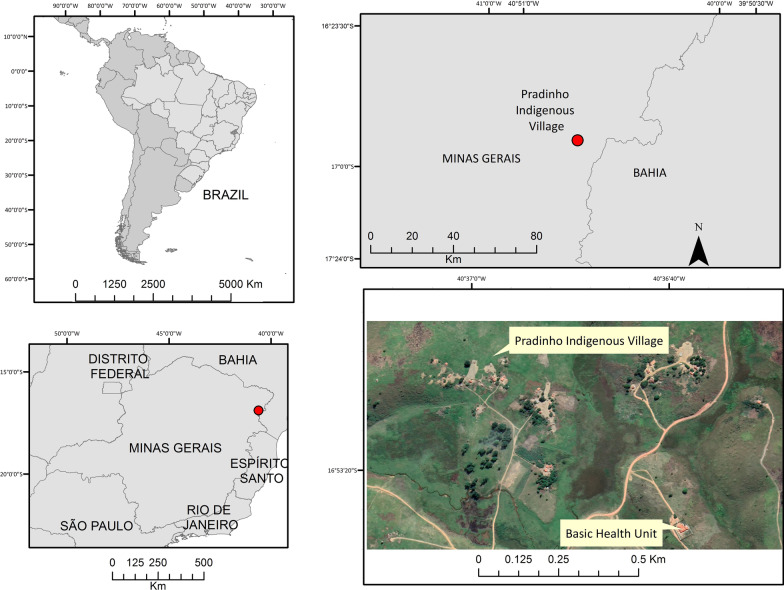

## Background

Human rabies is a neglected reemerging disease that has a major public health impact in poor communities and underdeveloped countries [1]. With high lethality, leading to death in almost 100% of those affected worldwide, rabies has an incidence of approximately 59,000 cases per year in 150 countries [2]. More than 99% of human rabies deaths occur in Asia and Africa as a result of an infected dog bite [3]. With the control of canine rabies in several Latin American countries, cases transmitted by vampire bats have increased considerably, with Brazil, Peru, Mexico and Colombia being the countries with the highest number of human rabies cases in the region, concentrating the highest number of cases in Peru and Colombia [4]. Epidemiological studies on outbreaks of human rabies in indigenous individuals are scarce in the world literature. The few published report records of at least 20 suspected cases of human rabies transmitted by vampire bats in 18 children and 2 adults between February and July 2011 in the Aguaruna indigenous tribe in the Imaza district, Peruvian Amazon region [5]. Another reports an outbreak that occurred in indigenous communities in the Ecuadorian Amazon in November 2011, reporting 11 deaths, nine of which were children under 15 years of age [6].

Human rabies outbreaks mediated by bats in Brazil are concentrated in the Amazon region and in the Northeast. In Bahia, between 1991 and 1992, in Aporá there were 3 fatal victims, and in Conde 2 people died [7]. In Pará, in 2004, a total of 21 people died (15 cases in Portel and 6 in Viseu). Still in Pará, in 2005, there were 15 cases in the municipality of Augusto Corrêa. In the state of Maranhão, in the municipalities of Turiaçu, Godofredo Viana, Carutapera and Cândido Mendes, 25 fatal cases were reported [8, 9]. In Amazonas, in 2017, in a riverside community in the municipality of Barcelos, the disease affected three siblings under 18 years of age, with a history of exposure to bats [10]. In Pará, in 2018, in the riverside area of the municipality of Melgaço, 9 out of 10 affected were also under 18 years old, all with a history of spoliation by bats [11].

**Fig. 1:**
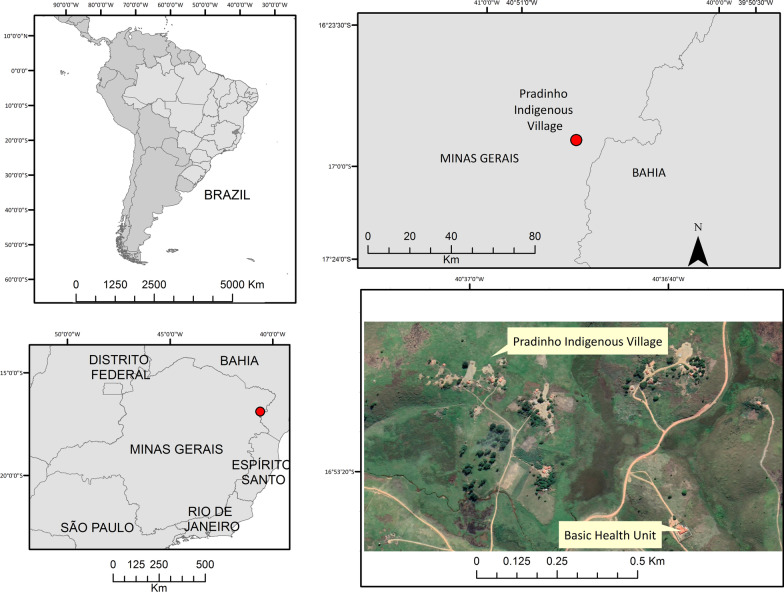
The geographic location of the Pradinho indigenous village, Bertopolis, Minas Gerais, Brazil

The last cases of human rabies that occurred so far in Minas Gerais were registered in 2005 in the municipality of Prados, and in 2012 in the municipality of Rio Casca [12]. Between April 4th and May 27th, 2022, 4 cases of human rabies were reported in Maxakali indigenous children related to exposure to bats in the Pradinho village, municipality of Bertópolis, Minas Gerais, Brazil [13] (Fig. 1). The Maxakali are used to living with domesticated cats and dogs and taming wild animals such as monkeys and bats. Due to this, aggressions are frequent and often go unnoticed by the health services in the region, a fact that represents a serious problem for sanitary and environmental control [14]. The Maxakali also have the particularity of maintaining their mother language, rites and customs, as Portuguese is spoken by few men. Communication between women and children is done through their own language, which has made it extremely difficult to understand the circumstances of exposure to potentially rabid animals for a more effective approach by health services [13, 14].

Considering the complexity of a human rabies outbreak in a Brazilian region characterized by a deficient health structure, added to the peculiarity of the geographical, linguistic, cultural and socioeconomic barrier of this indigenous population, we aimed to describe the epidemiological characteristics of this public health emergency.

## Case presentation

**Fig. 2:**
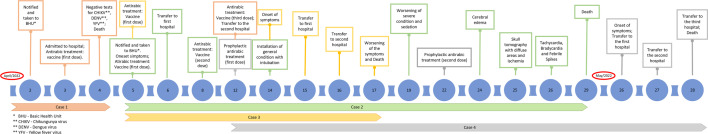
Timeline describing the four cases of rabies in indigenous children

We describe four cases of rabies among indigenous children that occurred in the indigenous village of Pradinho, municipality of Bertópolis, Minas Gerais, Brazil (Fig. 1). Cases were notified between April and May 2022. Pertinent information to each case is presented in the timeline below (Fig. 2).

### Case 1

**Fig. 3:**
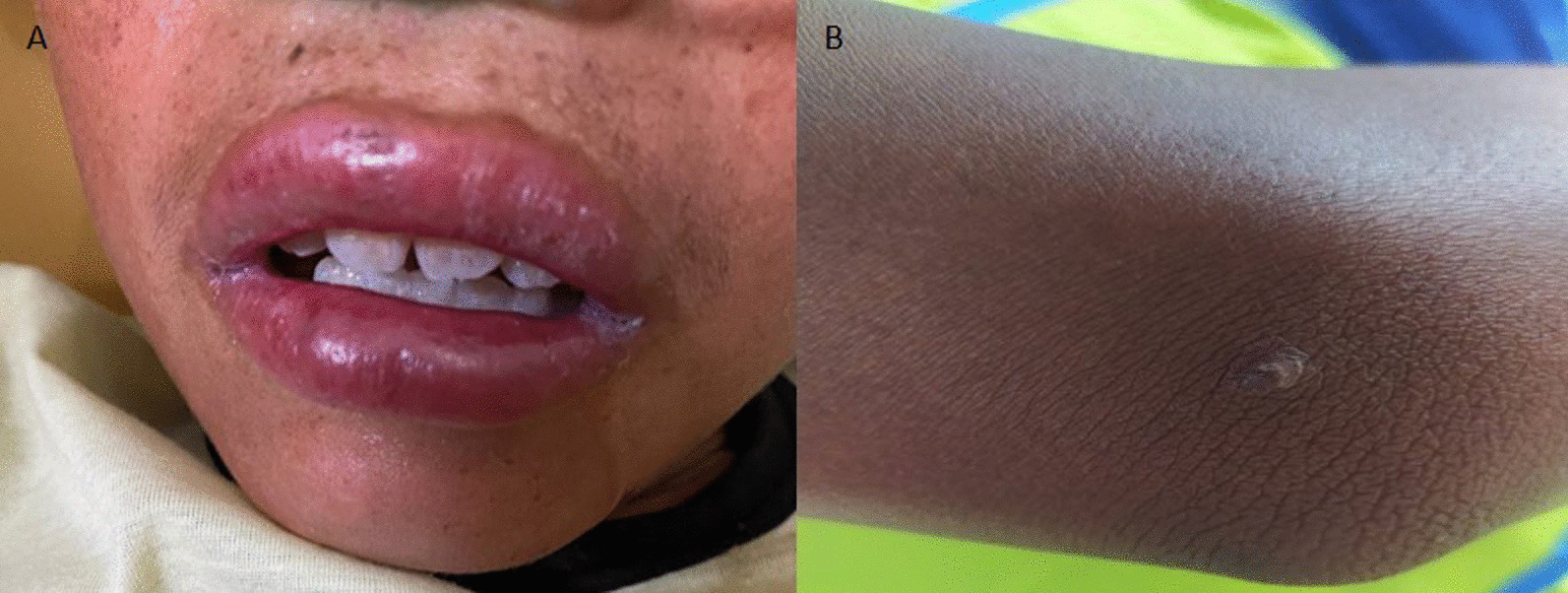
Location of the lesions caused by a bat bite on the children: **A** Case 1: upper lip; **B** Case 2: right elbow

A 12-year-old boy, resident in Maravilha Village, with a superficial bite on the upper lip by a bat that was not captured, approximately 10 days ago (Fig. 3A). He was taken to the Basic Health Unit on April 2nd, 2022, with fever, agitation, asthenia, sweating, paleness, lack of appetite, local pain, dyspnea and headache. On April 3rd, he was admitted to the hospital with a temperature of 38.5 °C, respiratory rate (RR) of 28 rpm, blood pressure (BP) of 120/70 mmHg, oxygen saturation (SaO_2_) of 88% and heart rate (HR) of 107 bpm. Due to the worsening of the clinical condition, he was transferred to an emergency unit with better structure in another city, where he received the first and only dose of post-exposure human rabies vaccine and 4.2 ml of immunoglobulin. On April 4th, samples of cerebrospinal fluid, saliva and blood were collected for diagnostic purposes. He had leukocytosis (24,720 mm^3^), increased oxaloacetic transaminase enzymes (125 U/ml) and creatine kinase (2894 U/ml). There was clinical worsening with vomiting, agitation, fever, sialorrhea, mydriasis, torpor, drop in saturation, bloody oral and nasal discharge, muscle spasms, seizures, hypotension, being intubated, progressing to cardiorespiratory arrest and death. On April 8th, human rabies was confirmed by RT-PCR in cerebrospinal fluid and saliva. Identification of the AgV3 viral variant was based on genetic sequencing in a saliva sample.

### Case 2

A 12-year-old girl, resident in Nova Village, with a history of a single superficial bat bite on the right elbow (Fig. 3B), presented with fever and headache. On April 6th, 2022, she was transferred to a hospital unit with persistent fever, BP of 147/85 mmHg, SaO_2_ of 99%, HR of 80, RR of 22 rpm, glucose of 107 U/L, carbon dioxide pressure (pCO_2_) reduction of 31 mmHg and alkaline phosphatase of 294 U/L. She underwent three doses of post-exposure anti-rabies vaccine on April 5th, 8th and 12th. On April 6th, she received 2.8 ml of immunoglobulin. On April 13th, cerebrospinal fluid and saliva were collected for diagnostic conclusion. On April 14th, when her clinical condition worsened (sweating, difficulty breathing, tachycardia and fever), she was intubated and treated with amantadine 100 mg every 12 h and with sapropterin 100 mg every 8 h enterally, as established by the adapted Milwaukee Protocol. On April 19th, she had sialorrhea and no reflexes. On the same day, the diagnosis of human rabies was confirmed by viral detection in the cerebrospinal fluid by RT-PCR, and the result of genetic sequencing in a saliva sample was compatible with a vampire bat lineage (AgV3). On April 22nd, there was a worsening of the clinical picture with tachycardia, hypotension and septic shock. On April 24th, immunoglobulin was administered. On April 25th, a CT scan of the skull showed a loss of differentiation between the white and gray matter of the brain and diffuse areas of ischemic distress. On April 26th, the severe condition persisted, with tachycardia, bradycardia and fever spikes, and on April 29th, she died.

### Case 3

A five-year-old boy, resident in Maravilha Village, with no history of a bat bite. On April 5th, 2022, he received the first and only dose of pre-exposure anti-rabies vaccine because he was a family member and had intense recreational contact with the child in case 1 who died. Symptoms started on April 14th with cough, anorexia, listlessness, dehydration and bloody diarrhea. He was transferred to hospital on April 15th, with HR of 127 bpm, RR of 26 rpm and SaO_2_ of 96%; malnourished, coughing and distended abdomen; evolving with pallor, dyspnea, fever, alteration in the level of consciousness, cyanosis of the extremities; seizures; sialorrhea; sweating; and tachycardia. After clinical worsening on April 16th, he was transferred to a reference hospital, presenting new symptoms: drowsiness, lack of appetite, drop in O_2_ saturation and seizures. Oxygen therapy was administered and laboratory examination revealed leukocytosis. On April 17th, he evolved with a general worsening with convulsive crises, muscle spasms, lowered level of consciousness, drop in O_2_ saturation and fever spikes. Laboratory tests identified hypoglycemia (29 mg/dL), increased urea (83 mg/dL) and leukocytosis (17,400 mm^3^) with a continuous increase in the percentage of segmented neutrophils (80%). On April 17th, he progressed to cardiac arrest and death. On the same day, CSF, oropharynx and hair follicle samples were collected for diagnosis. On April 26th, human rabies was laboratory confirmed through PCR viral detection in such specimens collected post mortem. It was not possible to detect the viral variant due to insufficient biological material.

### Case 4

A four-year-old girl, resident in Nova Village, with no history of biting, licking or scratching and no defined date of exposure, being a relative of the adolescent in case 2. She received two doses of pre-exposure anti-rabies vaccine on April 12th and 22nd, 2022. On May 26th, she started showing symptoms, including muscle weakness and loss of consciousness. She was referred to the hospital without verbal reaction or motor stimuli, with non-reactive pupils, dry skin and small lesions on her upper limbs. She showed vital signs with SaO_2_ of 59%, HR of 126 bpm; glucose of 226 mg/dL and temperature of 35.6 °C. On May 27th, she was transferred to another hospital with diarrhea, prostration, loss of movement, Glasgow 3, blood glucose of 437 mg/dL, hypothermia, without pain reactions or verbal stimuli. On May 28th, she was transferred to a reference hospital in a very serious condition, presenting with unconsciousness, Glasgow 3, SaO_2_ of 60%, non-palpable peripheral pulses, hypothermia, being intubated. Evolving to death one hour after admission. Soon after, a saliva sample was collected for diagnostic definition. Rabies diagnosis was only confirmed on June 10th by PCR detection in post mortem saliva. However, it was not possible to detect the viral variant due to insufficient biological material.

## Discussion and conclusions

This study describes four cases of human rabies in indigenous people, accidentally transmitted by an unidentified bat species, with a viral variant of the vampire bat.

Transmission occurred among children aged 4 to 12 years, and at least two cases were related to each other, although they lived in different households. The Maxakali people have customs that make them susceptible to rabies. They domesticate bats, venerating them as a divine symbol in their religious rituals [13]. They live in areas with precarious infrastructure and sanitary conditions, limitations in communication with non-indigenous people due to the use of the native language; and access to information about possible disease transmission mechanisms is impaired [15].

Of the cases of human rabies that occurred among the Maxakali, only case 2 was confirmed by ante-mortem laboratory tests. CSF, saliva and hair follicle samples from the nape of the neck of hospitalized patients were tested. All samples were sent to the Fundação Ezequiel Dias Laboratory (FUNED) in Belo Horizonte, which forwarded them to the Pasteur Institute in São Paulo, which is a national reference. Although direct immunofluorescence is considered the gold standard for the diagnosis of rabies, this was done using the reverse transcriptase reaction, followed by the polymerase chain reaction (RT-PCR) of the hair follicle and saliva [16]. Biological samples of serum and nasopharynx were collected and sent to the FUNED for differential diagnosis. The lack of acceptance by the family members for the post mortem brain sample collection was responsible for its non-performance. Soon after death, the Indians immediately claim the body for the mourning ritual. It was only possible to proceed with the sequencing and identification of the viral variant in the first two cases through molecular biology in the saliva, as in the others, the collection of material was insufficient, unlike the outbreaks in Viseu and Portel, where all the strains of rabies virus isolated of the 12 human CNS samples were antigenically typed [17].

The incubation period was estimated only in case 1 (10 days). Case 2 did not have a defined exposure date. The others involved indirect contact with the bat. The average incubation period for rabies in humans is 45 days; in children, it may be shorter due to body variables (size and weight), site of exposure and immune status [18]. Between the first symptoms and death, there was an average of 7.7 days. This period differs from that observed in the outbreak in Turiaçu, MA, which ranged from 16 to 39 days [19].

The signs and symptoms were similar to those of the outbreaks in Turiaçu, Portel and Viseu, compatible with the paralytic neurological phase associated with the virus transmitted by bats [17, 19]. The therapeutic approach varied between cases, but converged with the worsening of the clinical picture. Despite all the palliative treatment, it was not possible to avoid the death of any patient, contrary to what happened in the outbreak in Barcelos-AM, in which a teenager was cured, representing the second case of a patient who survived the disease in Brazil, the first case occurred in 2008, in the state of Pernambuco. In both cases, the adapted Milwaukee Protocol was used, also known as the Recife Protocol, a reference to the capital of the State of Pernambuco, where the successful treatment took place. This protocol consists of coma induction and administration of antivirals [10].

After the children were hospitalized, even after the administration of immunoglobulin and vaccine, there was a rapid worsening of the clinical condition of cases 1, 3 and 4, requiring the adoption of therapeutic measures for the maintenance of the patients. It was necessary to keep the patients isolated, intubated, with ventilatory support, peripheral and central venous access, indwelling urinary catheter, nasogastric tube, sedation and symptomatic medication. Management after laboratory confirmation of rabies requires continuous or intermittent monitoring of the patient in the intensive care unit with extensive work-up of clinical and laboratory tests to maintain the patient's hemodynamic stability [20].

Perhaps because she was the only patient to be submitted to the adapted Milwaukee Protocol [21], case 2 had the longest survival time compared to the others. Other factors that may have contributed to this may be the greater distance between the port of entry and the central nervous system, in addition to all the antiviral therapy received during the prodromal period of the disease. In general, the delay in prophylaxis may have contributed to the lethality, perhaps due to the health team's lack of experience in recognizing the signs and symptoms of the prodromal period.

Despite the immune response being able to produce neutralizing agents from 14 days after the first dose of the pre-exposure prophylaxis (PrEP) regimen [22], we cannot state that case 4 could have been avoided if the child had received such regimen when case 2 was reported, since there is a possibility that the patient’s previous state of health is immunocompromised at the time of application. The precarious nutritional conditions and basic sanitation that are characteristic of this population [15] may have interfered with the effectiveness of the anamnestic response that culminated in the inability to eliminate the circulating virus, rapidly progressing to encephalitis and, consequently, to death.

With the confirmation of the cases and the intense contact of this population with bats, a PrEP scheme was established with 2 doses of human anti-rabies vaccine (0 and 7 days) of 0.5 ml intramuscularly for the entire Maxakali population, contrary to what happened in the Melgaço—PA outbreak, in which all those involved did not receive post-exposure prophylaxis (PEP) or PrEP [11]. Vaccination was offered by the health team to the entire Maxakali population at strategic points and through an active search for missing persons. In addition, a four-dose PEP regimen (0, 3, 7, and 14 days) of 40 IU equine rabies immunoglobulin (ERIG) per kg or 20 IU human rabies immunoglobulin (HRIG) per kg body weight was administered to all contacts of confirmed cases. In higher risk situations, ERIG is the first choice for passive immunoprophylaxis and immunoglobulin should be used in cases of immunosuppressed patients and when there is contraindication to the use or lack of heterologous serum [23]. The 37 direct contacts were transported to the Cura D'Ars Hospital in Machacalis for the administration of the aforementioned prophylactic scheme, in accordance with the recommendation of the Ministry of Health and the WHO [3, 23].

In addition, surveillance, control and disease prevention measures were carried out after the outbreak in the focal region, such as anti-rabies vaccination of dogs and cats, active search for contact persons, capture of hematophagous bats, treatment of bats with vampiric paste to control the species, collection and analysis of samples of bats found dead or fallen to carry out direct immunofluorescence (DIF) for rabies, epidemiological investigation in the region to verify spoliation of bats in production animals, presence of epizootics or report of animals with clinical signs, implementation of health education actions to sensitize the village population about the risk of transmitting rabies and train local health professionals about the importance of active surveillance to prevent new cases [24].

Despite the challenges, it was possible through the use of the One Health approach to mobilize various sectors, disciplines and communities to intervene in complex problems of the human, animal and environmental interface of this population. This approach made it possible to strengthen rabies monitoring, surveillance and reporting systems at the regional, national and local levels to prevent and detect re-emergences in this population [25].

The outbreak in question has some similarities with other outbreaks that occurred in Brazil and South America, as they mainly affected children exposed to bats, in low-income regions, riverside populations, indigenous peoples, with low population density, lack of electricity, vulnerable housing and difficult access to health services [26].

In addition to the low social indicators, the Pradinho community has experienced in this emergency the challenge of the language barrier imposed by the dependence on interethnic contact that often hinders timely access to the health team and the unavailability of PEP, available only in a municipality 37 km away from distance.

This is the first rabies outbreak in an indigenous population in Brazil whose cases were caused by bats of unknown species. Furthermore, most cases evolved with paralytic rabies, with at least one case showing agitation, indicating an atypical disease presentation.

## Data Availability

The datasets generated during and/or analyzed in this study are available from the corresponding authors on reasonable request.

## Abbreviations

AM: Amazonas State (*in Portuguese*)
BPM: Beats per minute
BP: Blood pressure
CHIKV: Chikungunya virus
CT: Computed tomography
DENV: Dengue virus
DIF: Direct immunofluorescence
ERIG: Equine rabies immunoglobulin
FUNED: Fundação Ezequiel Dias (*in Portuguese*)
HR: Heart rate
HRIG: Human rabies immunoglobulin
IU: International unit
MA: Maranhão State (*in Portuguese*)
MmHg: Millimetre (s) of Mercury
PA: Pará State (*in Portuguese*)
pCO_2_: Carbon dioxide pressure
PEP: Post-exposure prophylaxis
PrEP: Pre-exposure prophylaxis
RR: Respiratory rate
RPM: Respirations per minute
RT-PCR: Reverse transcription polymerase chain reaction
SaO_2_: Oxygen saturation
BHU: Basic Health Unit
WHO: World Health Organization
YFV: Yellow fever virus
